# The Use of a Mechanical Non-Penetrating Captive Bolt Device for the Euthanasia of Neonate Lambs

**DOI:** 10.3390/ani8040049

**Published:** 2018-04-02

**Authors:** Andrew Grist, Jeff A. Lines, Toby G. Knowles, Charles W. Mason, Stephen B. Wotton

**Affiliations:** 1 School of Veterinary Sciences, University of Bristol, Langford House, Langford, Bristol BS40 5DU, UK; toby.knowles@bristol.ac.uk (T.G.K.); steve.wotton@bristol.ac.uk (S.B.W.); 2 Silsoe Livestock Systems, Wrest Park, Silsoe, Bedford MK45 4HR, UK; jeff.lines@silsoeresearch.org.uk; 3 Humane Slaughter Association, The Old School, Brewhouse Hill, Wheathampstead, Hertfordshire AL4 8AN, UK; charlie@hsa.org.uk

**Keywords:** animal welfare, euthanasia, livestock, mechanical killing, on-farm killing, neonate lamb

## Abstract

**Simple Summary:**

No stockperson or producer of lamb wants or likes to euthanase their young animals (neonates). At present, there are few reliable methods of achieving a humane dispatch of a neonate lamb should it be required. In the United Kingdom, the main method used in these cases is manually impacting the head by swinging the animal against a hard surface or hitting the animal with a hard object such as a hammer. This paper examines the use of a blank cartridge powered device to stun-kill neonate lambs immediately (i.e., before the animal can feel the application). Using this method, a suitable application point and power of cartridge has been determined, providing the stockperson with a reliable and repeatable single application method for euthanasing young lambs without the animal feeling any pain, as the device produces brain death faster than the nerve impulse can travel to the brain. This will improve animal welfare on-farm in these circumstances.

**Abstract:**

A non-penetrating captive bolt device, powered by a 1-grain 0.22″ cartridge delivering a calculated kinetic energy of 47 Joules was tested as a euthanasia method on 200 neonate lambs *(Ovis aries)* of 4.464 kg (SD (Standard deviation) ± 1.056) mean dead weight, to assess effectiveness and shot position. Every lamb (n = 200) was effectively stunned when the weapon was applied powered by a brown, 1-grain cartridge but 10/200 (5%) of the lambs displayed rhythmic or agonal breathing and were subsequently euthanased using euthatal (Merial, UK, GTIN: 03661103015550). Evaluation of the method was conducted using behavioural indicators of brain dysfunction followed by post-mortem examination of the heads. A second trial was conducted using a higher velocity 1.25-grain cartridge and a specific shot position on 48 lambs (mean dead weight = 6.21 kg, SD ± 1.24) averaging 5 days old. One hundred percent of the lambs in the second trial were immediately stun-killed. Given this complete kill rate and the sample size of the study, the study provides a statistical 95% confidence interval of 92.6% to 100%. The use of the Accles & Shelvoke “CASH” Small Animal Tool (Birmingham, UK) can therefore be recommended for the euthanasia of neonate lambs with a 1.25-grain cartridge and a specific shooting position.

## 1. Introduction

An inevitable consequence of modern production techniques is that occasionally the stockperson will be faced with the problem of euthanasia of young lamb for various reasons, such as production efficiencies (e.g., males born to a milking herd) and seriously sick young livestock that are beyond treatment. Usually, the choices that are available to them are either leaving the neonates with their mothers in the hope that they may recover, or the use of “casualty killing”. Casualty or surplus killing of young animals on-farm is usually carried out by administering a blow to the head, which is generally performed by swinging the young animal against the floor or a wall, or an impact to the head with a weighted device such as a hammer [1]. Although widely used as a means of casualty killing, the effectiveness of this method is heavily dependent on the strength and skill of the operator and, consequently, the probability of achieving an immediate kill in all cases is low. Furthermore, a lack of proper training and human error can lead to pain and distress to the animal and in addition it has to be considered that gaining proficiency in this method will take a variable period of time, during which there is the potential for animal welfare to be severely compromised. These methods are also usually aesthetically unpleasant for both the operator and any bystanders.

The Humane Slaughter Association (HSA) carried out a survey in 1996 [1], to look at the culling methods used for young lambs and piglets. The results showed that the majority of young sick lambs are left to die, whilst a manual blow to the head was the normal method applied to casualty piglets. The majority of respondents were not satisfied with their current method of casualty or surplus slaughter and all of them expressed an interest in an alternative device. At the time of the survey, there were no alternative methods available for killing young livestock. 

In a previous DEFRA study (United Kingdom Department for Environment, Food and Rural Affairs, MH0116), an assessment of 240 lambs showed that there was a significant difference between the number of animals showing signs of rhythmic breathing when using different devices. The study also showed that the Accles & Shelvoke small animal tool (Birmingham, UK) was the most effective at producing a stun/kill using behavioural assessments. The trials (MH0116) were conducted using a pink 1.25-grain cartridge in the Accles & Shelvoke small animal tool and demonstrated that the gun produced an effective stun/kill with lambs (n = 80). In preparation for the current study, we were informed (Accles & Shelvoke) that the current model of the Accles & Shelvoke small animal tool was only proofed for use with brown 1-grain cartridges, and therefore we were initially unable to change to a higher strength cartridge should animals survive the treatment with a brown 1-grain cartridge.

Captive bolt devices are used widely for the humane stunning and killing of adult livestock. The device used in this study (i.e., the Accles & Shelvoke “Cash” Small Animal Killer, CPK 200) was specifically designed for the killing of large birds and has also been demonstrated, using behavioural indicators, to be suitable for the euthanasia of neonate pigs and goats [2,3], with the use on the latter species requiring a specific shot position. Finnie, et al. [4] found that the application a non-penetrating captive bolt gun to 4 to 6 week-old lambs produced sufficient traumatic brain injury to suggest that it is an acceptable method of euthanasia. In addition, Sutherland, et al. [5] demonstrated the success of a non-penetrating captive bolt gun to result in immediate insensibility and death verified by EEG assessment in neonate goats up to 48 hours of age.

This paper reports the methods and findings of DEFRA project MH0150 to investigate the effectiveness of the Accles & Shelvoke “Cash” Small Animal Killer (CPK 200), powered by a brown 1-grain cartridge, to produce a humane stun/kill in 200 neonate lambs that were approximately ≤8 days old in trial one and a second trial of 48 neonate lambs that were approximately ≤8 days old using the Accles & Shelvoke “Cash” Small Animal Killer (CPK 200) powered by a pink 1.25 grain cartridge. The trial also sought to ascertain the most effective shot position to ensure an immediate stun/kill. The weapon and its action have been described elsewhere [2]. As there are various terms used for the termination of livestock including casualty slaughter, casualty killing, dispatch, culling etc., we will, within this paper refer to ‘euthanasia’ as best describing an immediate stun/kill in terms of brain function.

## 2. Materials and Methods

### 2.1. Method Trial 1

**Figure 1:**
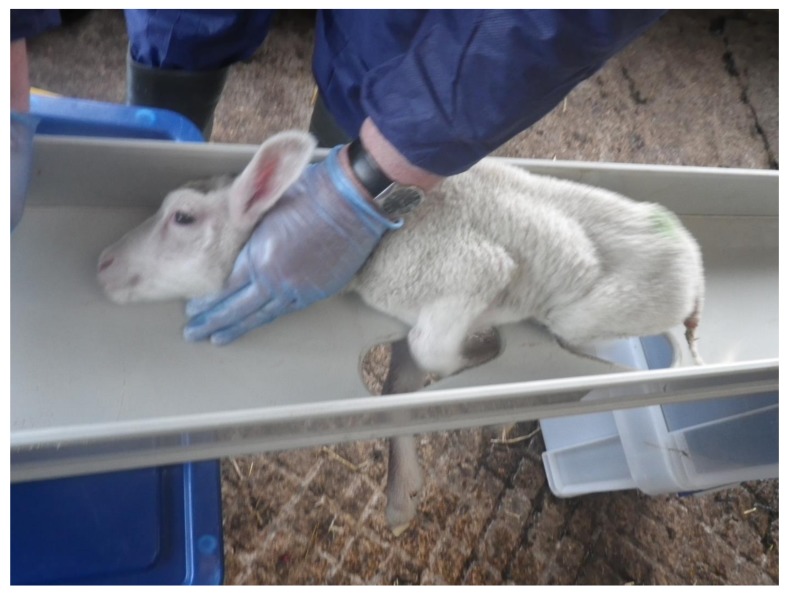
Lamb held in a restrainer prior to euthanasia with light pressure.

A total of 200 *Ovis aries* lambs (mean dead weight = 4.464 kg, SD = ±1.056), a mixture of Friesland and Suffolk crossbreeds, were allocated to trial one. These animals were healthy male lambs that were surplus to the requirement of a dairy-sheep farm and would normally have been euthanased on-farm. The lambs (≤8 days old) were restrained in a non-flexible plastic restrainer that was designed and built by Bock Industries (Philipsburg, PA, USA) [6] in collaboration with the University of Bristol (Figure 1). Following restraint, the animals were shot with the Accles & Shelvoke small animal tool, with a 1-grain cartridge as the power source producing a calculated kinetic energy of 47 Joules.

In trial one, the shot position was systematically altered between animals to assess the required shot position to achieve a stun/kill. The shot position was initially applied in the recommended shot position for sheep when using a penetrating captive bolt gun, i.e., the highest central point of the head aiming straight down towards the angle of the jaw, and more dorsal positions were systematically applied in the event of an animal displaying any signs of continued brain function.

Following application of the shot, the animal was assessed for behavioural signs of brain function [2,3,7,8] for a period of three minutes, with any animals displaying agonal breathing being assessed for a longer period. Any animals displaying behavioural signs of continued brain function were euthanased by an injection of pentobarbital (‘Euthatal’, Merial, UK GTIN: 03661103015550). Signs assessed were as follows:

1. The absence of rhythmic breathing—which is controlled by structures within the medulla oblongata and innervated by the reticular activating system [8].
2. The absence of a positive corneal reflex—a reflex with a neural pathway that passes adjacent to and partially through the reticular formation [8].
3. The absence of a positive palpebral reflex—a brainstem reflex.
4. The absence of response to painful stimuli (needle prick to the nose)—a cortical arc reflex [8].

The animals were also subjectively assessed for post-shot movement (clonic activity), based on the criteria given in Table 1, and the time from shot to cessation of this activity was recorded.

Once assessment was completed and the brain death of the lamb was confirmed using the absence of the behavioural signs of continued brain function described above, a sequentially numbered ear tag of kill order was attached and the lamb placed in a correspondingly numbered bag to be hard frozen pending post-mortem examination.

### 2.2. Method Trial 2

Following consultation with the gun manufacturers, a pink 1.25-grain cartridge was acquired and a further field trial on 50 lambs was undertaken to evaluate the performance of the gun at a higher velocity. The lambs (mean dead weight = 6.21 kg, SD = ±1.24), averaging 5 days old, were restrained in the Bock restrainer and shot with the Accles & Shelvoke small animal tool with a pink 1.25-grain cartridge producing a calculated kinetic energy of 107 Joules. The shot position selected for trial two was between the ears, with the chin tucked into the neck as described by Sutherland, et al. [5] and by Grist, et al. [3] for neonate goats.

The animals were assessed post-shot using the same descriptors as in trial one.

Once assessment was completed and the death of the lamb was confirmed, a sequentially numbered ear tag was attached and the lamb was placed in a bag to be hard frozen pending post-mortem examination.

### 2.3. Post Mortem Examination of Heads

**Figure 2:**
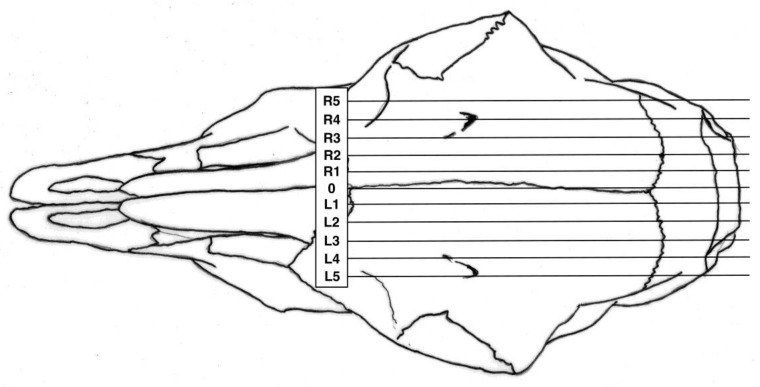
Lateral target area, with 0 representing the midline based on the suture of the paired frontal bones, L figures being to the left and R figures being to the right of this midline.

**Figure 3:**
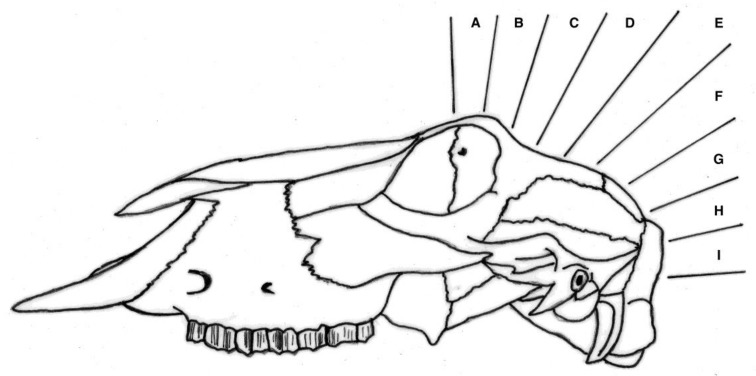
Sagittal targeting areas using surface topography with F representing a shot on the frontal-parietal suture, G on the parietal bone, H on the parietal-occipital suture etc.

All experimental animals were frozen after killing and subsequently thawed for post-mortem examination. The head was examined for external lesions including laceration at the point of impact. For all carcasses, the skin from the head was removed following a T incision cranial to the shoulders and extending forward to the nose. The impact site was photographed with a digital camera (PENTAX Optio WG-1 or PENTAX K-50, Ricoh Imaging Europe, Rungis, France) before removal of any haematoma and the periosteum, in order to expose fracture lines extending from the impact site. Photographs were taken of the fracture patterns to allow for later comparison. To determine the most effective application position, the location of each application was recorded retrospectively during post mortem analysis, based on the lateral and sagittal guide (Figure 2 and Figure 3), in order to produce an application code for each head. The heads were removed from the carcass and subsequently individually hard frozen in sequentially numbered bags with the corresponding ear tag to facilitate sectioning on the sagittal plane for photography of cranial and brain lesions to be undertaken. Hard freezing reduces the frictional distortion of the brain during sectioning. 

Each head was removed from the bag once the number had been noted and the ear number was checked to ensure correlation. The head was split on the sagittal plane using an electric band saw (Startrite Meat Master, UK) and both sides were photographed on the medial plane with a digital camera and post-mortem findings recorded. 

**Figure 4:**
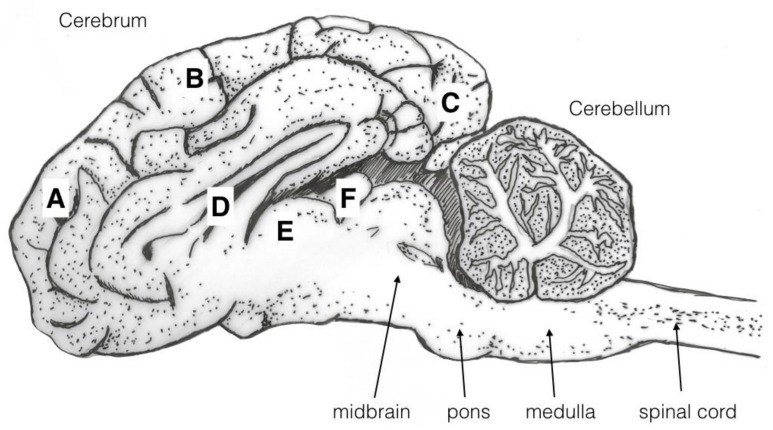
Diagram of a sagittal section of a brain illustrating the areas examined for macroscopic damage. Areas are labelled as frontal cerebrum (A), parietal cerebrum (B), occipital cerebrum (C) and lateral ventricle (D). These were scored on the basis of 0 = no damage, 1 = slight deformation, 2 = moderate deformation and 3 = severe deformation of the area. The frontal (A), parietal, and (B) occipital cerebrums (C), lateral ventricle (D), thalamus (E), pineal gland (F), midbrain, pons, medulla and cerebellum were assessed for the presence or absence of haemorrhages.

The macroscopic brain lesions were assessed by one of the authors (AG), subjectively and blind to the weight ranges and carcass numbers, utilising a scale adapted from Sharp, et al. [9] and also used in Grist, et al. [2,3,5] where 0 = no damage, 1 = slight deformation, 2 = moderate deformation and 3 = severe deformation of the area. The areas examined for macroscopic damage were the frontal, parietal and occipital cerebrums including the structure of the lateral ventricle and the cerebellum as detailed in Figure 4. The maximum possible total brain damage score was thus 15. The frontal, parietal and occipital cerebrums, lateral ventricle, thalamus, midbrain, pineal gland, pons, medulla and cerebellum were assessed for the presence or absence of haemorrhaging, with a score of 1 indicating the presence of haemorrhage and 0 the absence of haemorrhage, giving a maximum possible total haemorrhage score of 10. 

The shot position for each animal was also recorded based on lateral and sagittal targeting, as seen in Figure 2 and Figure 3. These data were recorded in an Excel database (Microsoft) for statistical analysis.

### 2.4. Statistical Analysis

We report summary statistics below. The joint effects of variables such as carcass weight, and those describing the shot position and damage on the outcome variable ‘time to cessation of movement’ was investigated using a general linear model (GLM). Because only two values were actually recorded for the movement score the variables effecting movement score were investigated using a binary logistic regression. Predictor variables were simply fitted as main effects and the residuals checked to ensure that the models met the required assumptions. An independent *t*-test was used to test for differences between groups of lambs. Histograms of the variables were used to check that a parametric approach to testing was appropriate. IBM SPSS Statistics v23 was used for all analyses. To facilitate detailed statistical analysis, the shot code for each head was converted to a number to produce a lateral and ventral position score. Using this method, the lateral score L1 became −1, L2 became −2 etc., with right ventral positions being designated as positive scores. The ventral targeting position was converted to a numerical targeting score with A being converted to 1, B to 2, C to 3, etc.

This work was approved by the University of Bristol’s Ethical Committee and carried out under United Kingdom Home Office Licence (PPL 30/2999 and PPL 30/3404).

## 3. Results

### 3.1. Trial 1

Every lamb (n = 200) was effectively stunned when the weapon was applied, powered by a brown 1-grain cartridge, but 10/200 (5%) of the lambs displayed rhythmic or agonal breathing and were euthanased using euthatal. The first lamb to display agonal breathing was shot a second time, which again proved to be unsuccessful at killing the animal.

An independent samples *t*-test showed that the time to loss of movement was the only variable that was significantly different between the lambs that were euthanased with euthatal (not killed) and those that were effectively stun/killed (*t* = 5.17, *p* ≤ 0.01). Those that were euthanased with euthatal (not killed) displayed loss of movement at 83.8 s (SE (Standard Error) = 13.99) and those that were effectively stun/killed had a mean time to loss of movement of 141.79 s (SE = 2.47). There was a trend for the ventral shot position score to differ between the two groups with a mean for those killed of 4.73 (SE = 0.096) and those not killed of 3.60 (SE = 0.562) (*t* = 1.986, *p* = 0.077). The 10 lambs that were effectively stunned but not killed by the treatment were removed from further data analysis (leaving n = 190 lambs).

**Figure 5:**
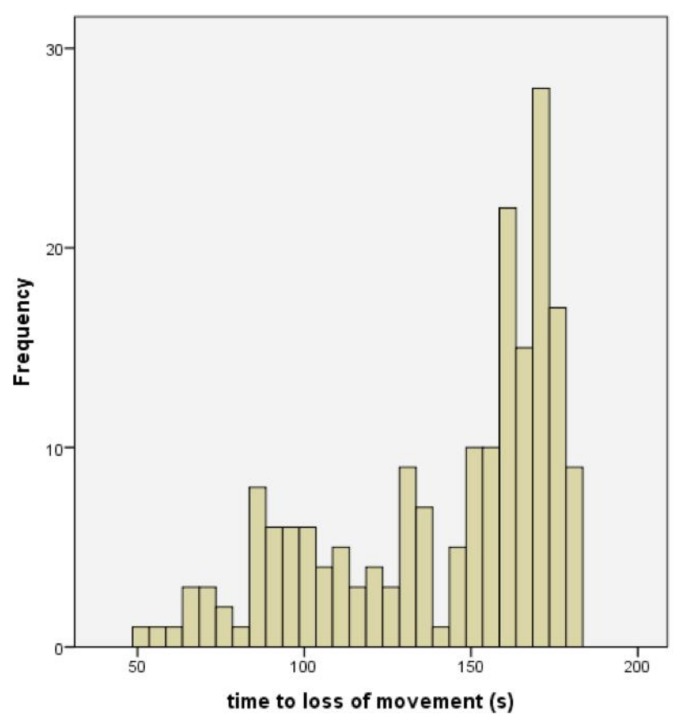
The distribution of time to loss of movement in lambs post-shot (mean = 141.79, SD = 34.084, n = 190).

Figure 5 shows the time to loss of movement, for the remaining 190 lambs.

#### 3.1.1. Factors Related to “Time to Loss of Movement”

**Figure 6:**
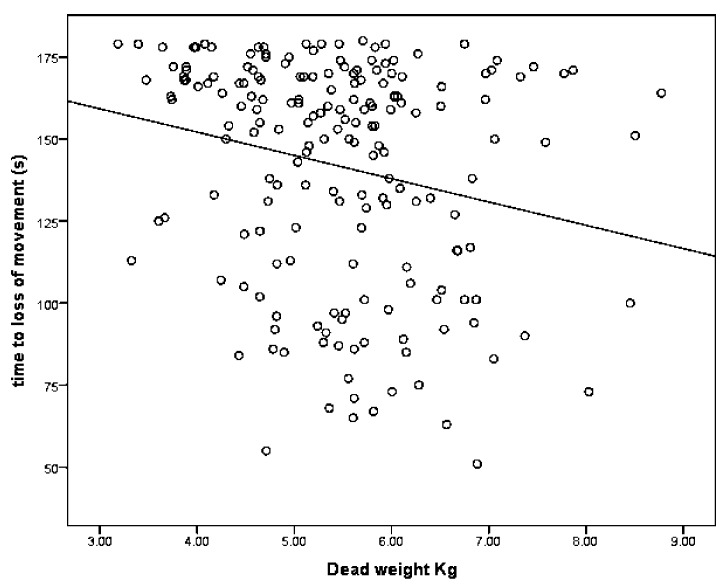
The effect of dead weight on time to loss of movement in lambs.

**Figure 7:**
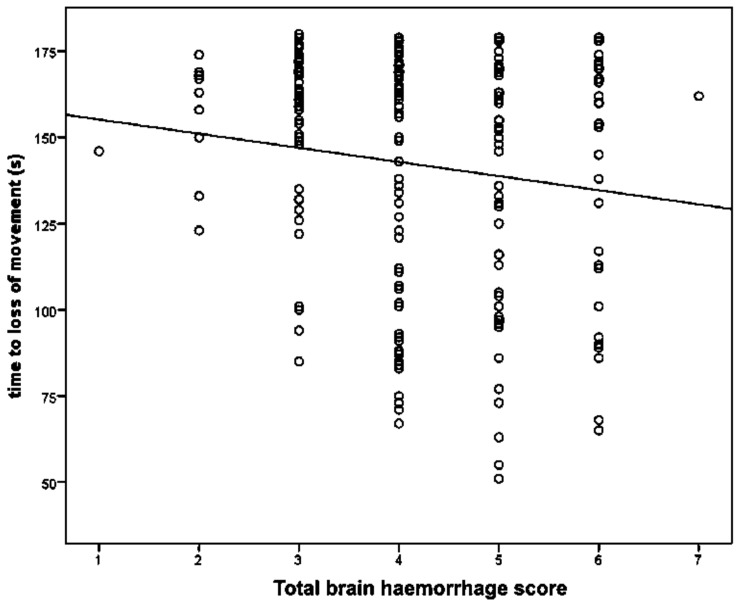
The effect of total brain haemorrhage score on time to loss of movement in lambs, post-shot.

The parameter estimates from the GLM analysis of “time to loss of movement” are shown in Table 2. These data demonstrate that there was no significant effect of total damage score or lateral shot position score. There was a significant effect of dead weight on ‘time to loss of movement’. On average, for every additional kilogram of dead weight, there was a decrease in time to loss of movement of 7.8 s (Table 2 and Figure 6). There was a significant effect of total haemorrhage score with each unit increase in score associated with a 4.8 s decrease in time to loss of movement (Table 3 and Figure 7), and also a significant effect of ventral shot position score with each unit increase in score associated with a 4.0 s increase in time to loss of movement.

#### 3.1.2. Factors Related to ‘Movement Score’

The parameter estimates from a binary logistic regression of ‘movement score’ differentiating between the scores of 2 and 3 are shown in Table 3. There was no significant effect of total damage score, total haemorrhage score or lateral shot position score on the probability of a movement score of either 2 or 3. There was a significant effect of dead weight and ventral shot position score. With increased dead weight, there was an increased probability of a movement score of 3 (Odds Ratio = 2.629), and with increased ventral score there was a decreased probability of a movement score of 3 (Odds Ratio = 0.546). Mean dead weight was 4.98 (SE = 0.114) kg for those with a score of 2, and 5.71 (SE = 0.093) kg for those with a score of 3. Mean ventral position score was 5.13 (SE = 0.139) for those with a score of 2, and 4.51 (SE = 0.123) for those with a score of 3.

#### 3.1.3. General Description of Post-Mortem Findings

**Figure 8:**
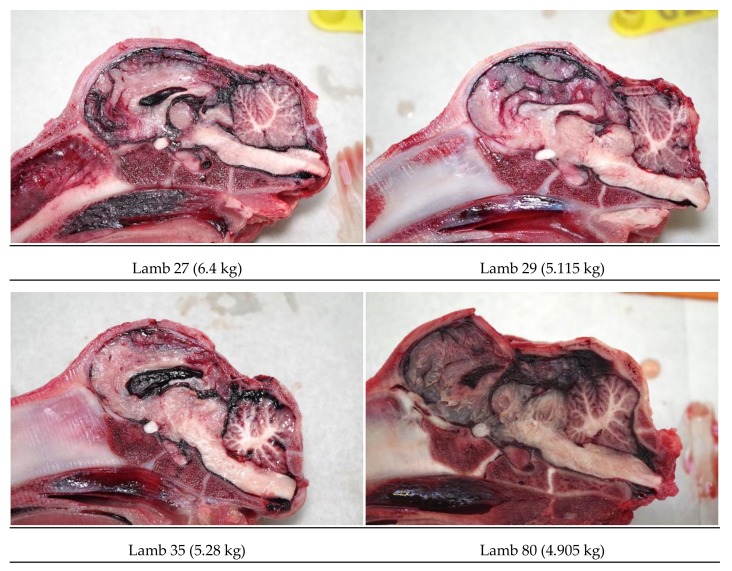
Examples of post-shot trauma, medial sagittal section.

All animals displayed a depressed fracture of the cranial plates and concurrent subdural haematoma corresponding to the impact footprint of the convex head of the bolt. Fracture of the cranial plates was a common finding, however the deviation of the plates into the brain was less than was observed with piglets [2,7]. The lesions within the brain were dependent on the shot position with macroscopic lesions occurring under the application position. Parenchymal haemorrhages were evident within the thalamus, frontal parietal and occipital lobes of cerebrum and the cerebellum (Figure 8).

#### 3.1.4. Shot Position

**Figure 9:**
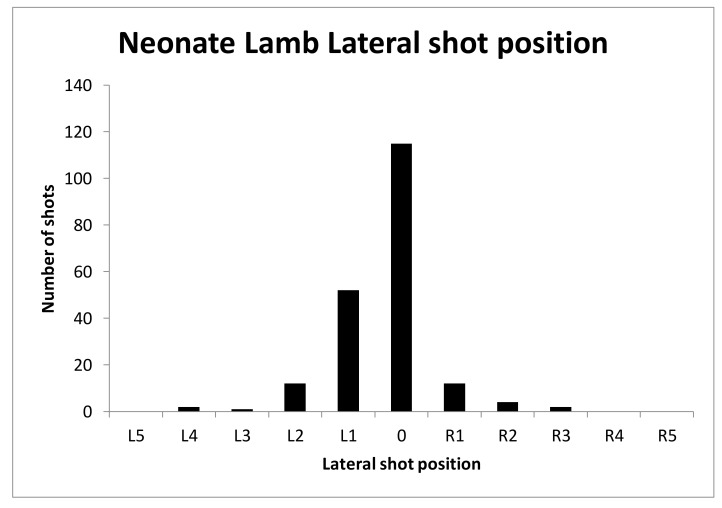
Graph of lateral shot position over the 200 lambs subjectively scored based on the lateral targeting position indicator in Figure 2 with 0 being on the midline, L1 being 0.5 cm to the left of this line, L2 1 cm to the left of the midline etc. and the codes R being to the right of the midline viewing the head from a caudal position.

**Figure 10:**
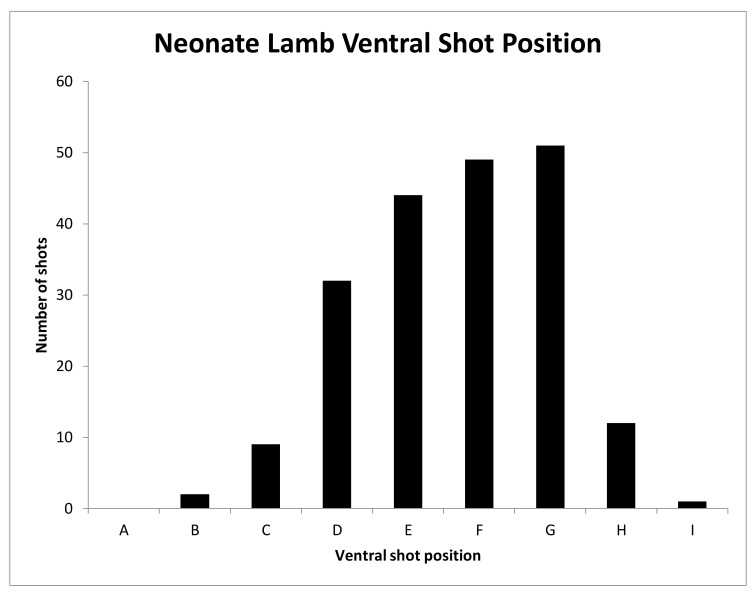
Graph of ventral shot position over the 200 lambs subjectively scored based on the ventral targeting position indicator in Figure 3.

**Figure 11:**
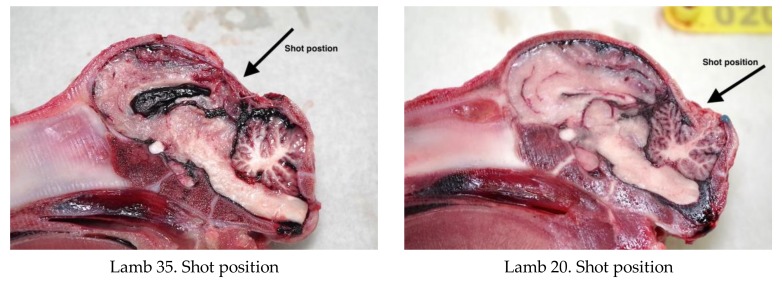
Variation in shot position and macroscopic brain damage in lamb head sagittal sections.

The shot positions applied to neonate lambs were varied deliberately (Figure 9, Figure 10 and Figure 11) to try to identify a position that would be 100% effective. Unfortunately, we were unable to locate such a position using the CPK200 with brown 1-grain cartridge.

### 3.2. Trial 2

#### 3.2.1. General

**Figure 12:**
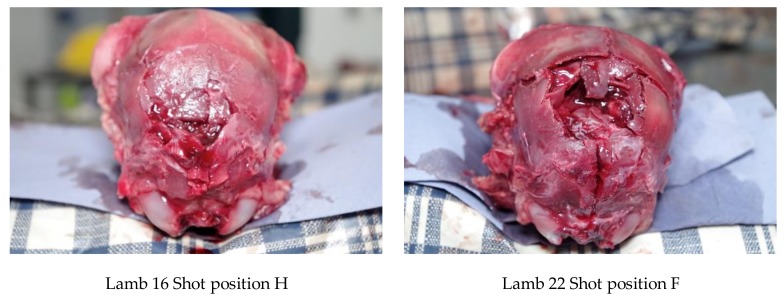
Comparative effects of sagittal shot position of neonate lambs, demonstrating greater fracturing with more cranial application.

The deadweight of the lambs averaged 6.21 kg (SD = ±1.24). Twelve lambs (24%) produced nasal bleeding following the shot, with a further 6 (12%) having a laceration to the head. All the lambs presented with a depressed fracture of the cranium, with those shot in a more lateral position (H) tending to have a less discrete lesion (Figure 12).

**Figure 13:**
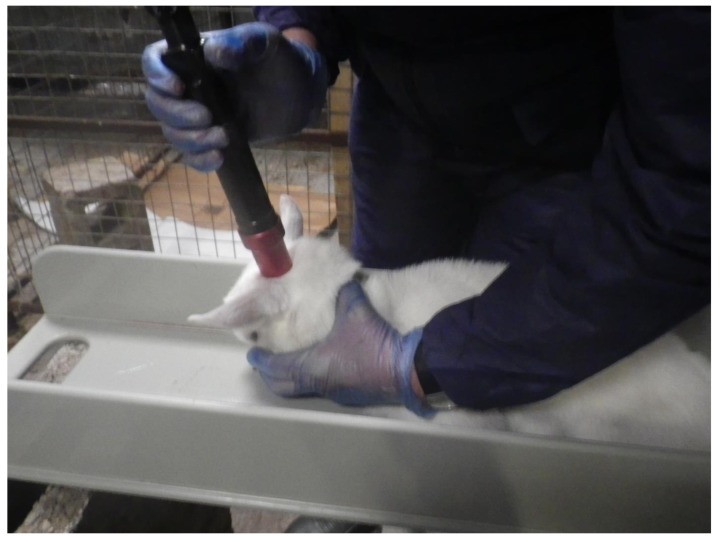
Revised shoot position for a neonate lamb. At the back of the head, on the midline, with the chin tucked into the neck, a similar position to that used for goats in Grist, et al. [3] and Sutherland, et al. [5].

One hundred per cent of lambs in the study (n = 48) were immediately killed when the dorsal shot position, as proposed for the euthanasia of goats [3,5] (Figure 13), was applied. Given this complete kill rate and the sample size of the study, a statistical 95% confidence interval provides a very maximum percentage of animals that would not immediately be stunned/killed by this mechanical non-penetrating captive bolt system, to be at most 7.4% and at least 0%.

#### 3.2.2. Factors Related to “Time to Loss of Movement”

**Figure 14:**
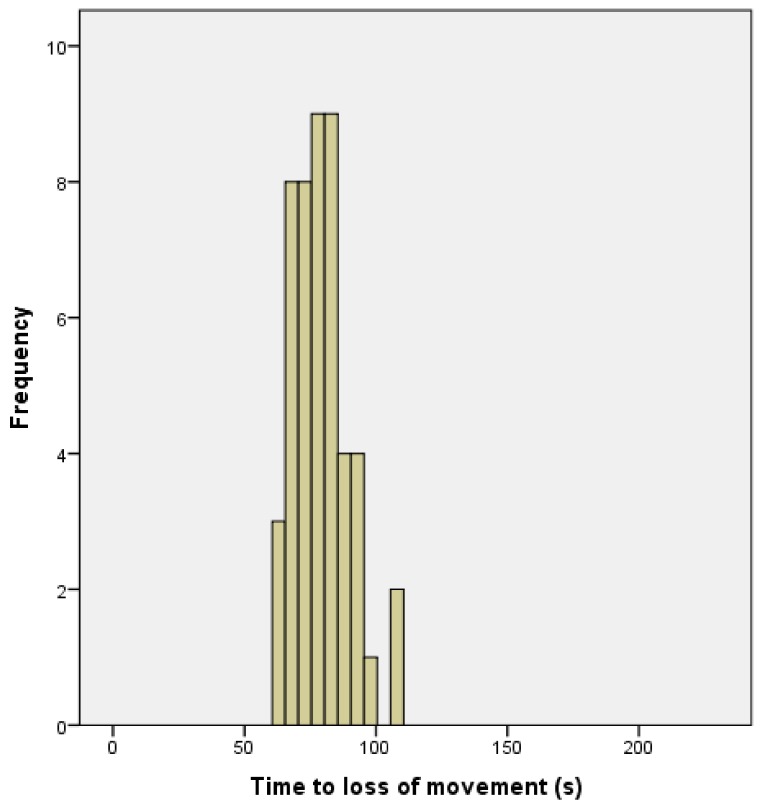
The distribution of time to loss of movement in lambs post-shot (mean = 79.42, SD = 10.657, n = 48).

**Figure 15:**
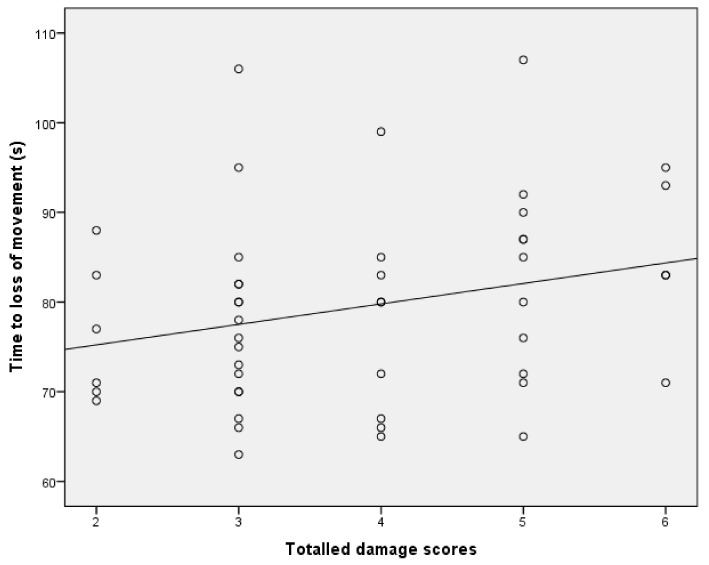
The “trend” for an effect (*p* = 0.071) of total macroscopic brain damage on time to loss of movement in lambs.

The time from application to loss of movement is shown in Figure 14. The parameter estimates from the GLM analysis of “time to loss of movement” are shown in Table 4. This shows that although there was no significant effect of total damage score on time to loss of movement (Figure 15), there was a trend (0.071) for such an effect. There were no other significant relationships.

#### 3.2.3. Shot Position

**Figure 16:**
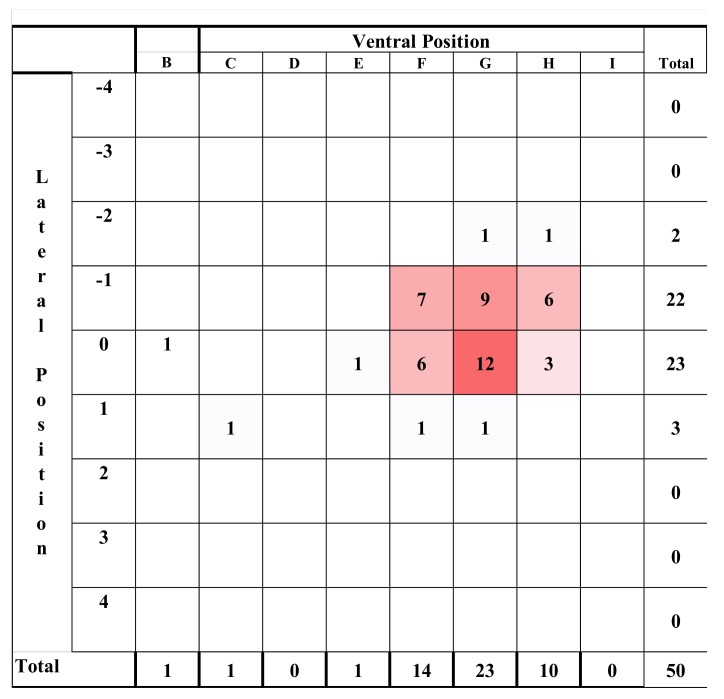
Heat map of the distribution of shooting positions for the 50 lambs. Lateral target area, with 0 representing the midline based on the suture of the paired frontal bones with positive values to the LHS and negative values to the RHS of the head (see Figure 8). Ventral targeting areas using surface topography with F representing a shot on the frontal-parietal suture, G on the parietal bone, H on the parietal-occipital suture etc., with C rostral to the head (Figure 9).

The first two lambs were shot in the parietal positions B:0 and C:R1, respectively. The second lamb had to be euthanased with 1 mL euthatal at 7 min due to the presence of rhythmic breathing. All further lambs were shot in the back of the head (E to H) as can be seen in Figure 16.

The first two lambs presented with damage consistent with a parietal shot, with the major damage being to the parietal and occipital cerebrums, and no damage to the cerebellum. The second lamb displayed no macroscopic haemorrhages in the region of the midbrain, pons and medulla.

**Figure 17:**
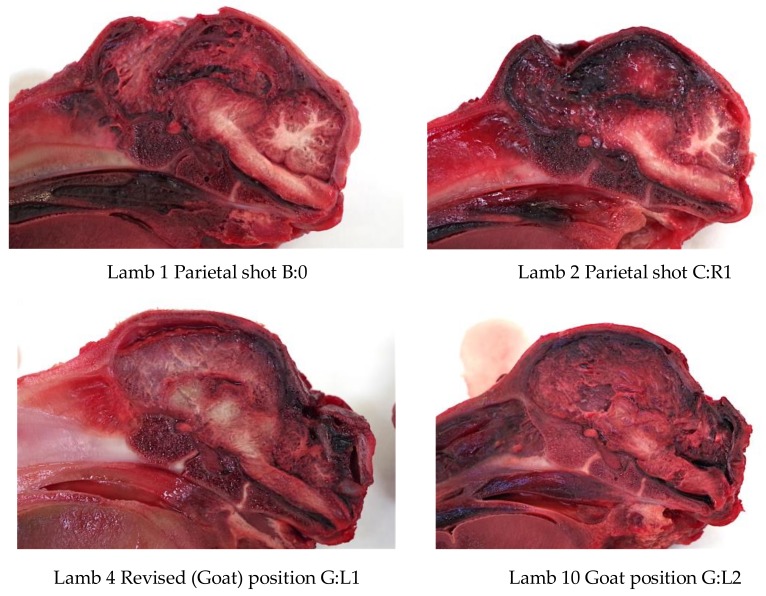
Trial 2 using 1.25-grain cartridges, variation in fracture indentation and brain haemorrhages based on application position.

All the lambs shot in the goat position (rear of head) presented with damage to the cerebrum and haemorrhages within the pons, medulla and midbrain. Examples of damage variation according to application position are demonstrated in Figure 17.

## 4. Discussion

By using visual evoked potentials, Daly and Whittington [10] determined that impartation of kinetic energy to the cranium via impact of the bolt was the main determinant of an effective stun in sheep. This was borne out in the present study where all the lambs in both trials were effectively stunned, as indicated by behavioural measures. 

Any method that disables brain function without simultaneously affecting the spinal cord will allow the latter to produce clonic activity. Gregory [11] stated that the loss of the modulatory control of the somatomotor cortex, demonstrated by convulsions, can be used as an indicator of early brain failure leading to death. In the case of mechanical stunning, this clonic activity has been associated with an isoelectric EEG [12,13]. Although this ‘uncontrolled physical activity’ post shot can be unsettling to the casual observer, once explained to the stockperson it can be used as an indicator of the success of the method in producing brain dysfunction. In trial one, the mean time to loss of movement was 141.79 s (SD = 34.084, n = 196), in trial two, the mean time to loss of movement in lambs post-shot was 79.42 s (SD = 10.657, n = 48). This reduction in movement time can be attributed to various factors including the increase in applied kinetic energy from 47 J to 107 J, the increase in cartridge loading from 1-grain to 1.25-grain, the larger animals in trial two (5.45 kg, SD = 1.056 in trial one and 6.29 kg, SD = 1.192 in trial two) and the increased subjective, macroscopic brain damage score in the second trial (damage score of trial 2 = 5.44, SD = 1.78, compared with damage score trial 1 = 3.86, SD = 1.21). That there was only a trend for an effect of total damage score and no other significant variables associated with time to loss of movement in the second trial was probably due to the smaller sample size in this supplementary trial (n = 50 trial 2 compared with n = 190 in trial 1) and the greater dead weight (6.29 kg, SD = 1.192 trial 2 compared with 5.45 kg, SD = 1.056 in trial 1). 

Gibson, et al. [14] found that damage to the thalamus, midbrain, pons, occipital and parietal lobe were associated with complete concussion leading to death. Finnie, et al. [4] and Terlouw, et al. [8] discuss the specific brain anatomy of sheep, in particular the effect of the tentorium cerebrelli in this species and the effect on cerebellum damage due to impact against this extension of the dura mater and the possibility of herniation through this as an added effect of a concussion wave. The shot position, recommended for goat kids [3,6], was found to be successful in this study and would direct the pressure waves directly at the tentorium and possibly directly down to the medulla and brainstem, thereby affecting the reticular activating system [6]. In this study, there was an effect of increased macroscopic total brain haemorrhage score, with each unit increase in score being associated with a 4.8 s decrease in time to loss of movement. 

Intermittent, agonal gasping is distinct from rhythmic breathing and is not an indicator of consciousness [15]. The possible causes of agonal breathing in cases of mechanical stunning are discussed in Grist, et al. [2,7] and Terlouw, et al. [15]. St John [16] reviewed the current theories of the origin of normal breathing (eupnoea) proposing that eupnoea requires the control of the pons and medulla, whereas gasping is due to the effects of a latent pacemaker mechanism initiated by severe hypoxia or ischaemia, or loss of pontile and rostral medullary influences.

This infers that the presence of agonal gasping, as long as it ceases, could be considered as an indicator that the force applied to the head has produced dysfunction in the pons and medulla, and therefore the reticular activating system and ascending reticular activating system, which demonstrates a level of unconsciousness that precedes brain death [8].

The results of our study, using the standard CASH small animal tool, powered by a brown cap 1-grain cartridge, showed that the gun delivered a concussive blow of sufficient energy to produce unconsciousness in lambs. However, the percussive blow delivered by the gun did not immediately kill ten (5%) of the two hundred lambs tested. It is proposed that the lambs that were not immediately killed had suffered severe cortical damage but insufficient damage to the brain stem to prevent the return of rhythmic breathing and other brain stem reflexes. Preliminary work carried out by Hewitt (Defra MH0116) has also suggested that the CASH Small Animal Tool was not always effective with lambs unless a higher strength cartridge (e.g., pink 1.25-grain) was used. Gibson, et al. [17] found a significant relationship between cartridge fill weight and peak velocity with the one grain cartridge and suggested that this variation may be due to the volumetric filling method of less nitrocellulose propellant with more silica filler.

As a precautionary measure, it is recommended that the use of a percussive stun-kill using a CASH Small Animal Tool with a brown 1-grain cartridge should not be used with neonate lambs. Although the lower strength cartridges (Accles & Shelvoke, 0.22 calibre, brown cap, 1-grain blank cartridge, 47 J) were found to produce an immediate stunned state, the use of behavioural indicators demonstrated this kinetic energy to be ineffective at immediately killing every animal. However, by increasing the energy of the impact by on average 60 Joules, the CASH Small Animal Tool produced an effective stun-kill in every animal that was shot in the rear of the head, the recommended shot position for goat kids [3,5]. The use of a pink 1.25-grain blank cartridge is not recommended by the manufacturer (Accles & Shelvoke) with the current weapon due to the damaging effect of this increased energy on the recuperating buffers. However, discussions with Accles & Shelvoke. have revealed that they are developing the Small Animal Tool to operate with higher energy cartridges that would meet the requirements suggested by this research. 

Given the size of the second part of this study (n = 48) and the evidence that every lamb was effectively stun/killed, statistical analysis gave a 95% confidence interval of 92.6% to 100%. However, these results are supported by the first part of this study albeit using a lower cartridge strength which resulted in an effective stun in all animals (n = 200) with 10 animals displaying some signs of life post-shot and the previous research investigating the use of non-penetrating captive bolt devices on poultry [18] and neonate livestock [2,5,7]. The use of the Accles & Shelvoke “CASH” Small Animal Tool can therefore be recommended for the euthanasia of neonate lambs with a 1.25-grain cartridge and a specific shooting position. As all animals were successfully stunned by this method, should the behavioural indicators demonstrate continued brain function following the application, the authors suggest that the operative is prepared to kill the animal by pithing, via the depressed fracture, cervical dislocation, bleeding or prolonged exposure to anoxia.

## 5. Conclusions

Based upon behavioural indicators of brain death, the Accles & Shelvoke “CASH” Small Animal Tool is an effective single shot euthanasia device for neonate lambs, provided that a specific shot position is used, that is, on the midline at the back of the head with the chin tucked in, in conjunction with a 1.25-grain cartridge.

## Figures and Tables

**Table 1:** Subjective scoring system used to assess post-stun/kill movement based on the level of spinal reflex activity, ranging from 0 (no activity post-stun) to 3 (gross uncontrolled physical movement).

Score	Descriptor	Description
0	No activity	Very little movement.
1	Mild activity	Some mild uncontrolled physical movement of limbs.
2	Moderate activity	Considerable uncontrolled physical movement of the limbs.
3	Severe	Gross uncontrolled physical movement.

**Table 2:** Parameter estimates from the general linear model (GLM) testing for an effect of dead weight (kg), total damage score, lateral and ventral shot position score and total haemorrhage score on time to loss of movement (s).

Parameter	B	Std. Error	*t*	Sig.
Intercept	182.162	18.245	9.984	0.000
Dead Weight (kg)	−7.776	2.306	−3.372	0.001
Total	0.655	1.486	0.441	0.660
Lateral Position Score	0.746	2.815	0.265	0.791
Ventral Position Score	4.026	1.941	2.075	0.039
Total Haemorrhage Score	−4.767	2.158	−2.209	0.028

**Table 3:** The parameter estimates from a binary logistic regression testing the effect of variables on movement scores of 2 or 3. The regression was coded with the score of 3 as the positive (logistic) outcome.

Parameter	B	Std. Error	*t*	Sig.	Exp(B)
Intercept	−1.490	1.371	1.180	0.227	0.225
Dead Weight (kg)	0.967	0.202	22.989	≤0.001	2.629
Total	−0.120	0.108	1.220	0.269	0.887
Lateral Position Score	−0.122	0.199	0.378	0.539	0.885
Ventral Position Score	−0.606	0.149	16.624	≤0.001	0.546
Total Haemorrhage Score	0.111	0.156	0.512	0.474	1.118

**Table 4:** Parameter estimates from the GLM testing for an effect of total damage score on time to loss of movement (s).

Parameter Estimates						
Dependent Variable: Time to Loss of Movement (s)						
Parameter	B	Std. Error	*t*	Sig.	95% Confidence Interval	
					Lower Bound	Upper Bound
Intercept	70.665	4.971	14.214	0.000	60.658	80.672
Total Damage	2.283	1.236	1.846	0.071	−0.206	4.772
